# Impact of stress hyperglycemia mediating tissue-level collaterals on futile recanalization in large vessel occlusion patients

**DOI:** 10.3389/fendo.2025.1682343

**Published:** 2025-12-16

**Authors:** Xinyu Li, Junling Fu, Liping Huang, Jin Liu, Shuyu Jiang, You Wang, Chen Gong, Tao Xu, Yangmei Chen

**Affiliations:** 1 Department of Neurology, The Second Affiliated Hospital of Chongqing Medical University, Chongqing, China; 2 Department of Neurology, Chongqing University Three Gorges Hospital, Chongqing, China; 3 Key Laboratory of Major Brain Disease and Aging Research (Ministry of Education), Chongqing Medical University, Chongqing, China

**Keywords:** stress hyperglycemia ratio, tissue-level collaterals, large vessel occlusion, endovascular treatment, futile recanalization

## Abstract

**Background:**

The stress hyperglycemia ratio (SHR) is associated with unfavorable functional outcomes in patients with large vessel occlusion. The potential effect of SHR on tissue-level collaterals (TLC) and futile recanalization is not clear.

**Methods:**

This is a multicenter retrospective cohort study of patients with consecutive acute ischemic stroke due to large vessel occlusion (AIS-LVO) receiving endovascular treatment (EVT). The included patients had baseline glucose/HbA1c measurements and underwent cerebral perfusion imaging. TLC were measured using the hypoperfusion intensity ratio (HIR) [the volume ratio of brain tissue with (*T*_max_ > 10 s/*T*_max_ > 6 s)]. SHR was calculated as blood glucose (mmol/L)/[1.59 × HbA1C (%) − 2.59]. Using multivariable regression and mediation analyses, we determined the association among SHR, the TLC status, and futile recanalization (90-day modified Rankin Scale scores 3–6 despite successful recanalization).

**Results:**

A total of 246 patients met the inclusion criteria. Patients in the highest tertile of SHR were significantly more likely to suffer futile recanalization compared with those in the lowest tertile [adjusted OR (aOR) = 3.56, 95%CI = 1.73–7.30, *p* < 0.001]. The TLC (aOR = 3.38, 95%CI = 1.23–9.27, *p* = 0.018) was worse in patients with elevated SHR and also acted as an independent predictor of futile recanalization (aOR = 2.31, 95%CI = 1.32–4.05, *p* = 0.003). Mediation analyses showed that the increased SHR was associated with worse TLC, accounting for 9.7% (95%CI = 1.9%–28.0%) of the harmful effect on futile recanalization. Mediation analyses also indicated a partial mediation effect of the baseline larger ischemic core (effect value = 13.5%, 95%CI = 3.1%–32.0%).

**Conclusion:**

An increased SHR is correlated with unfavorable TLC and is associated with futile recanalization after EVT. Future prospective studies should independently validate our findings.

## Introduction

Patients with acute ischemic stroke due to large vessel occlusion (AIS-LVO) have poor prognosis, half of whom suffering from disability or mortality despite successful recanalization, termed futile recanalization (1, 2). Hyperglycemia at admission is associated with worse functional prognosis and with symptomatic intracranial hemorrhage in patients with AIS-LVO who received endovascular treatment (EVT) (3). However, four previous clinical randomized controlled trials (RCTs) failed to demonstrate the therapeutic effect of glucose-lowering therapy in patients with general acute ischemic stroke (4–7). This contradiction suggests that absolute hyperglycemia may be an insufficient marker and that the relative stress hyperglycemia ratio (SHR), which adjusts for the preexisting glycemic status via HbA1c, may more accurately capture the pathological stress response and serve as a superior prognostic biomarker (8, 9).

The underlying mechanisms by which stress hyperglycemia leads to poor outcomes, particularly in the context of futile recanalization, remain incompletely understood. Stress hyperglycemia arises from a complex interplay of counterregulatory hormones and inflammatory cytokines (10). Animal studies have suggested that this state can exacerbate microvascular thrombo-inflammation, impair reperfusion, and precipitate neurovascular injury following recanalization (11). We hypothesize that impaired tissue-level collaterals (TLC), a key factor in microcirculatory failure, may be a critical mediator of this process (12). TLC can be automatically quantified on perfusion imaging using the hypoperfusion intensity ratio (HIR) and is strongly associated with infarct progression and the no-reflow phenomenon (13).

While both SHR and TLC are independently linked to outcomes, the potential causal pathway connecting them has not been investigated. Specifically, whether SHR contributes to futile recanalization by adversely affecting TLC is unknown. Therefore, this study aimed to explore the association among stress hyperglycemia (measured by the SHR), TLC, and futile recanalization in patients with AIS-LVO, utilizing mediation analysis to test the hypothesis that TLC is a significant mediator.

## Methods

The data that support our results are available from the corresponding author upon reasonable request.

### Study design and patient selection

We performed a multicenter retrospective cohort study of consecutive AIS-LVO patients undergoing EVT at two comprehensive stroke centers (the Second Affiliated Hospital of Chongqing Medical University and Chongqing University Three Gorges Central Hospital) between January 2019 and September 2023. This retrospective investigation received ethical approval from the institutional review committees at both participating centers (no. 2024-93) and was conducted in accordance with the principles of the Declaration of Helsinki. Informed consent was obtained from each participant.

The inclusion criteria for this study were: 1) age ≥18 years; 2) diagnosis of AIS with LVO of the anterior circulation confirmed by digital subtraction angiography (DSA); 3) received EVT within 24 h of the estimated time of AIS-LVO; 4) completed baseline CT perfusion (CTP) examination before EVT, with high image quality; and 5) achieved successful recanalization by EVT, defined as Expanded Thrombolysis in Cerebral Infarction (eTICI) grades 2b–3. The exclusion criteria were: 1) insufficient data (fasting blood glucose or HbA1C) on the first day of stroke onset; 2) lack of at least one follow-up head non-contrast computed tomography (NCCT)/magnetic resonance imaging (MRI) within 48 h after EVT; 3) termination of EVT for technical reasons; 4) pre-stroke modified Rankin Scale (mRS) score >2; and 5) missed visit at subsequent 90-day follow-up (**Supplementary Figure S1**).

### Data collection

The demographic information and baseline clinical characteristics of all eligible patients were extracted, which included: 1) demographic information: age and sex; 2) medical history: smoking, drinking, hypertension, diabetes mellitus, hyperlipidemia, atrial fibrillation, coronary heart disease, ischemic stroke history, and cerebral hemorrhage history; 3) baseline characteristics: intravenous thrombolysis, blood glucose, HbA1C, and HIR (calculated as the volume ratio of brain tissue with *T*_max_ > 10 s/*T*_max_ > 6 s) (14); 4) severity of illness scores: pre-stroke mRS, the Trial of ORG 10172 in Acute Stroke Treatment (TOAST) classification, intravenous thrombolysis, baseline National Institutes of Health Stroke Scale (NIHSS) scores at admission, and the Alberta Stroke Program Early Computed Tomography Score (ASPECTS); and 5) surgical characteristics and intraoperative scores: the location of occlusion, passes of stent retriever, the time from stroke onset to groin puncture (OTP), the time from stroke onset to revascularization (OTR), the eTICI score on the final angiogram, and the American Society of Interventional and Therapeutic Neuroradiology/Society of Interventional Radiology (ASITN/SIR) collateral vessel grading system.

### Stress hyperglycemia assessment

The fasting blood glucose and HbA1c levels were measured in patient serum samples on admission. Fasting blood glucose was defined as the morning venous plasma glucose level measured after an overnight fast of at least 8 h, typically on the first morning following hospital admission. In this study, the SHR was utilized to quantify the extent of acute increases in the blood glucose levels in stressful situations. The SHR was calculated using the following formula: blood glucose (mmol/L)/[1.59 × HbA1C (%) − 2.59] (8). The SHR was analyzed both as a continuous variable and as a categorical variable based on tertiles. The continuous form was used to maximize the statistical power in the regression and mediation models, providing an odds ratio per 1-unit increase in SHR. All patients were divided into three groups based on the SHR tertiles in order to better describe the characteristics to visually inspect for a nonlinear dose–response relationship with the outcome—T1, T2, and T3—with the T1 group designated as the reference group.

### Outcomes

The mRS represents the levels of disability at 90 days [an mRS score from 0 (no symptoms) to 6 (death)] (15). The primary functional outcome was futile recanalization, defined as a 90-day mRS score of 3–6 despite successful recanalization (eTICI 2b–3) (16). Secondary functional outcomes included the distribution of the mRS score at 90 days (ordinal shift analysis), the proportion of patients without disability at 90 days (90-day mRS scores of 0–1), the proportion of favorable functional outcome at 90 days (90-day mRS scores of 0–3), and 90-day mortality (90-day mRS score of 6).

### Statistical analysis

In this study, we employed a complete-case analysis approach without missing key variables. The data presentation and analysis methods were determined by variable characteristics: continuous measures with normal distribution are expressed as the mean ± standard deviation (SD) and were analyzed using parametric tests (Student’s *t*-test for two groups and ANOVA for multiple groups), while the non-normally distributed continuous variables are reported as median (interquartile range, IQR) and were examined using non-parametric tests (Mann–Whitney *U* or Kruskal–Wallis). Categorical variables are presented as frequency percentages. Distribution normality was verified through Kolmogorov–Smirnov testing. The demographic, medical history, baseline characteristics, clinical variables, neuroimaging data, and outcomes were compared among groups.

Binary regression analysis was used to evaluate the association between SHR and clinical prognosis, adjusting for confounding variables. The final confounding factors included those with significance (*p* < 0.05) through univariable analysis and those associated with the outcomes in previous studies: model 1, which was unadjusted, and model 2, which was adjusted for age, occlusion site, baseline NIHSS, HIR, and OTR. Binary logistic regression was used to model the binary clinical outcomes adjusted for the above variables. For the distribution of the 90-day mRS scores (ordinal mRS shift), a multinomial ordinal logistic regression was applied to estimate a 1-point shift toward the lowered ordered value, indicating a better outcome.

Furthermore, cause mediation analysis was performed with the R *mediation* package to identify potential mediating mechanisms of the effect of SHR on futile recanalization. Only when the first three steps (i.e., steps a, b, and c) were satisfied could mediation be established in the fourth step (step c′). Four steps were performed using binary logistic regression tests adjusted for the variables in model 3. The predominant data flow that occurred during the disease progression at the risk factor and outcome levels was visualized with a Sankey plot using the R *ggplot2* and *ggalluvia* packages. To strengthen causal inference, all models within the mediation analysis framework were adjusted for a prespecified set of baseline potential confounders, which included age, occlusion site, the baseline NIHSS score, and the onset-to-reperfusion time (OTR).

Statistical analysis was performed using SPSS software (version 25.0; IBM SPSS Statistics) and R software (version 4.2.2). All tests were two-sided, and a *p*-value less than 0.05 was considered significant.

## Results

### Patient characteristics

A total of 246 patients who achieved successful recanalization were included in this study after excluding 66 patients with thrombectomy treatment more than 24 h after stroke onset, two patients with bilateral acute ischemic lesions, 42 patients with eTICI 0–2a, 137 patients who lacked data (HbA1c and FBG) on the first day of admission, and 367 patients without CTP (**Supplementary Figure S1**). The median age was 69 years (IQR = 58–76 years), the baseline NIHSS score was 13 (IQR = 10–18), and 138 (56.1%) patients were men. The median SHR for all included participants was 1.03 (IQR = 0.89–1.19). The 90-day futile recanalization and mortality rates were 48.0% and 19.1%, respectively (**Table 1**).

**Table 1:** Baseline characteristics and clinical outcomes according to groups categorized by stress hyperglycemia ratio (SHR) tripartite^a^.

Characteristics	Enrolled patients				*p*-value
	Overall (*N*=246)	T1 (*n*=82)	T2 (*n*=82)	T3 (*n*=82)	
Age (years), median (IQR)	69 (58–76)	69 (57–74)	66 (56–74)	72 (60–79)	0.004
Sex/male, *n* (%)	138 (56.1)	46 (56.1)	51 (62.2)	41 (50.0)	0.290
Medical history, *n* (%)					
Smoking	70 (28.5)	24 (29.3)	28 (34.1)	18 (22.0)	0.219
Drinking	72 (29.3)	18 (22.0)	31 (37.8)	23 (28.0)	0.079
Hypertension	139 (56.5)	43 (52.4)	48 (58.5)	48 (58.5)	0.661
Diabetes mellitus	42 (17.1)	17 (20.7)	9 (11.0)	16 (19.5)	0.195
Hyperlipidemia	33 (13.4)	13 (15.9)	14 (17.1)	6 (7.3)	0.136
Coronary heart disease	43 (17.5)	13 (15.9)	11 (13.4)	19 (23.2)	0.231
Atrial fibrillation	96 (39.0)	36 (43.9)	25 (30.5)	35 (42.7)	0.150
Ischemic stroke	32 (13.0)	10 (12.2)	12 (14.6)	10 (12.2)	0.866
Cerebral hemorrhage	3 (1.2)	1 (1.2)	1 (1.2)	1 (1.2)	1.000
Pre-stroke mRS, median (IQR)					0.204
0	232 (94.3)	78 (95.1)	75 (91.5)	79 (96.3)	
1	9 (3.7)	1 (1.2)	5 (6.1)	3 (3.7)	
2	5 (2.0)	3 (3.7)	2 (2.4)	0 (0.0)	
TOAST, *n* (%)					0.606
LAA	138 (56.1)	46 (56.1)	47 (57.3)	45 (54.9)	
CE	83 (33.7)	31 (37.8)	25 (30.5)	27 (32.9)	
Other	25 (10.2)	5 (6.1)	10 (12.2)	10 (12.2)	
Occlusion site, *n* (%)					0.548
ICA	79 (32.1)	29 (35.4)	26 (31.7)	24 (29.3)	
MCA-M1	126 (51.2)	44 (53.7)	40 (48.8)	42 (51.2)	
MCA-M2	41 (16.7)	9 (11.0)	16 (19.5)	16 (19.5)	
Baseline NIHSS score, median (IQR)	13 (10–18)	11 (8–16)	12 (9–18)	15 (11–19)	0.002
OTP (min), median (IQR)	380 (246–581)	416 (232–644)	344 (249–524)	387 (263–544)	0.671
OTR (min), median (IQR)	497 (338–723)	558 (328–583)	467 (323–655)	509 (406–688)	0.347
Intravenous_thrombolysis, median (IQR)	110 (44.7)	32 (39.0)	36 (43.9)	42 (51.2)	0.287
Glucose (mmol/L), median (IQR)	7.1 (6.0–8.6)	5.8 (5.3–6.7)	6.7 (6.3–7.4)	8.7 (7.7–10.3)	<0.001
HbA1C (%), median (IQR)	5.8 (5.5–6.3)	6.0 (5.6–6.6)	5.7 (5.5–6.1)	5.7 (5.3–6.1)	0.001
SHR, median (IQR)	1.03 (0.89–1.19)	0.85 (0.78–0.89)	1.03 (0.98–1.08)	1.29 (1.19–1.47)	<0.001
Primary outcome					
90-day mRS 3–6, *n* (%)	128 (52.0)	29 (35.4)	39 (47.6)	60 (73.2)	<0.001
Secondary outcomes					
90-day mRS, median (IQR)	3 (1-5)	2 (1-3)	2 (1-4)	4 (2-6)	<0.001
90-day mRS 0–1, *n* (%)	68 (27.6)	34 (41.5)	24 (29.3)	10 (12.2)	<0.001
90-day mRS 0–3, *n* (%)	159 (64.6)	64 (78.0)	59 (72.0)	36 (43.9)	<0.001
90-day mortality, *n* (%)	47 (19.1)	12 (14.6)	5 (6.1)	30 (36.6)	<0.001

*IQR*, interquartile range; *TOAST*, Trial of ORG 10172 in Acute Stroke Treatment; *LAA*, large artery atherosclerosis; *CE*, cardiogenic embolism; *ICA*, internal carotid artery; *MCA*, middle cerebral artery; *NIHSS*, National Institutes of Health Stroke Scale; *OTP*, time from stroke onset to groin puncture; *OTR*, time from stroke onset to revascularization; *mRS*, modified Rankin Scale.

a SHR tripartite: T1 (≤0.93), T2 (0.93–1.13), T3 (≥1.13).

**Table 1** exhibits the baseline characteristics of the patients according to the SHR tertiles. The enrolled patients were divided into three groups based on the SHR levels [tertile (T): T1 (≤0.93), T2 (0.93–1.13), and T3 (≥1.13)]. Patients in the highest tertile of SHR generally had a higher proportion of men (*p* = 0.004) and higher NIHSS scores (*p* = 0.002) compared with the lower tertile of the SHR group. Compared with individuals in the lower tertile of SHR, those in the higher tertile had a high proportion of futile recanalization (35.4% *vs*. 47.6% *vs*. 73.2%, *p* < 0.001) and 90-day mortality (17.2% *vs*. 19.3% *vs*. 34.5%, *p* < 0.001). For imaging details in **Table 2**, HIR and baseline ASPECTS showed significant differences among groups (p < 0.05). We further compared the differences between patients with futile recanalization and those without futile recanalization (**Supplementary Table S1**). Patients in the futile recanalization group were more likely to be older and women and to have higher blood glucose, higher HIR, and higher NIHSS scores and ASPECTS (*p* < 0.05).

**Table 2:** Presentation imaging details dichotomized by stress hyperglycemia ratio (SHR) tripartite^a^.

Characteristics	Enrolled patients				*p*-value
	Overall (*N*=246)	T1 (*n*=82)	T2 (*n*=82)	T3 (*n*=82)	
ASITN/SIR score, median (IQR)	1 (1–2)	1 (0–2)	1 (1–2)	1 (1–2)	0.742
HIR (volume ratio of brain tissue with *T*_max_ > 10 s/*T*_max_ > 6 s) (min), median (IQR)	0.33 (0.12–0.48)	0.26 (0.06–0.42)	0.29 (0.04–0.42)	0.42 (0.27–0.55)	<0.001
Baseline ASPECTS, median (IQR)	7 (5–8)	7 (6–8)	7 (5–8)	5 (3–8)	0.002

*IQR*, interquartile range; *ASITN/SIR*, American Society of Interventional and Therapeutic Neuroradiology/Society of Interventional Radiology; *HIR*, hypoperfusion intensity ratio; *ASPECTS*, Alberta Stroke Program Early CT Score.

a SHR tripartite: T1 (≤0.93), T2 (0.93–1.13), T3 (≥1.13).

### Impact of the SHR on functional outcomes

After adjusting for covariates, the T3 group had a higher proportion of futile recanalization than the T1 group [T3 *vs*. T1: adjusted OR (aOR) = 3.56, 95%CI = 1.73–7.30, *p* < 0.001]. However, there was no difference between the T1 and T2 groups (T2 *vs*. T1: aOR = 1.84, 95%CI = 0.94–3.62, *p* = 0.076) (**Table 3**). **Supplementary Table S2** shows the results of the binary logistic regression for futile recanalization adjusted for age, occlusion site, baseline NIHSS, HIR, and OTR.

**Figure 1:**
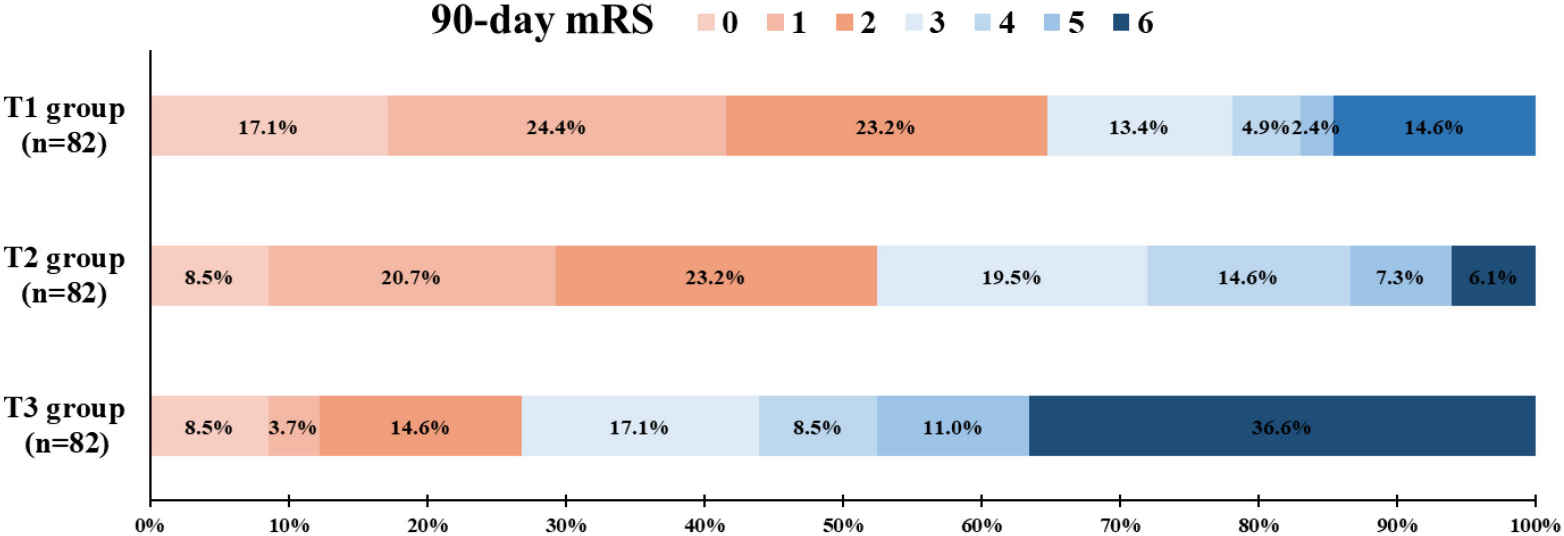
Distribution of the 90-day modified Rankin Scale (mRS) scores in the enrolled patients categorized by the stress hyperglycemia ratio (SHR). SHR tripartite: T1 (≤0.93), T2 (0.93–1.13), T3 (≥1.13).

**Figure 1** presents the distribution of the mRS scores at 90 days according to the SHR tertiles. In addition, a statistically significant shift toward a lower degree of functional disability in the 90-day mRS scores was observed, favoring the high over the lower tertile of SHR (*p* < 0.001). For other functional outcomes, patients in the T3 group had a lower proportion of 90-day mRS 0–1 (T3 *vs*. T1: aOR = 0.29, 95%CI = 0.12–0.67, *p* = 0.004; T3 *vs*. T2: aOR = 0.52, 95%CI = 0.26–1.03, *p* = 0.062) (**Table 3**). Similar results were observed in the adjusted models for the 90-day mRS 0–3 and mortality.

When SHR was a continuous variable, a higher SHR per unit was significantly associated with a higher risk of futile recanalization (aOR = 14.28, 95%CI = 4.39–46.47, *p* < 0.001), 90-day mRS (aOR = 13.99, 95%CI = 5.80–33.72, *p* < 0.001), mRS 0–1 (aOR = 0.09, 95%CI = 0.02–0.35, *p* < 0.001), mRS 0–3 (aOR = 0.06, 95%CI = 0.02–0.19, *p* < 0.001), and 90-day mortality (aOR = 11.54, 95%CI = 3.86–34.49, *p* < 0.001) (**Table 3**).

**Table 3:** Multivariable logistic regression analysis for clinical outcomes.

Category	Model 1		Model 2	
	aOR^*^ (95%CI)	*p*-value	aOR^*^ (95%CI)	*p*-value
Futile recanalization(90-day mRS 3–6)				
Continuous variable per unit	14.28 (4.39–46.47)	<0.001	7.82 (2.26–27.10)	0.001
Tripartite^a^				
T1 (*n*=82)	Reference		Reference	
T2 (*n*=82)	1.66 (0.89–3.10)	0.114	1.84 (0.94–3.62)	0.076
T3 (*n*=83)	4.98 (2.56–9.70)	<0.001	3.56 (1.73–7.30)	<0.001
90-day mRS				
Continuous variable per unit	13.99 (5.80–33.72)	<0.001	7.37 (2.99–18.17)	<0.001
Tripartite^a^				
T1 (*n*=82)	Reference		Reference	
T2 (*n*=82)	1.48 (0.86–2.54)	0.158	1.64 (0.94–2.84)	0.079
T3 (*n*=83)	5.02 (2.84–8.87)	<0.001	3.49 (1.93–6.30)	<0.001
90-day mRS 0–1				
Continuous variable per unit	0.09 (0.02–0.35)	<0.001	0.17 (0.04–0.74)	0.018
Tripartite^a^				
T1 (*n*=82)	Reference		Reference	
T2 (*n*=82)	0.58 (0.31–1.12)	0.104	0.52 (0.26–1.03)	0.062
T3 (*n*=83)	0.20 (0.09–0.43)	<0.001	0.29 (0.12–0.67)	0.004
90-day mRS 0–3				
Continuous variable per unit	0.06 (0.02–0.19)	<0.001	0.13 (0.04–0.43)	<0.001
Tripartite^a^				
T1 (*n*=82)	Reference		Reference	
T2 (*n*=82)	0.72 (0.35–1.47)	0.368	0.66 (0.31–1.43)	0.296
T3 (*n*=83)	0.22 (0.11–0.44)	<0.001	0.33 (0.16–0.70)	0.003
90-day mortality				
Continuous variable per unit	11.54 (3.86–34.49)	<0.001	6.03 (1.92–18.90)	0.002
Tripartite^a^				
T1 (*n*=82)	Reference		Reference	
T2 (*n*=82)	0.38 (0.13–1.13)	0.082	0.36 (0.11–1.17)	0.090
T3 (*n*=83)	3.37 (1.58–7.19)	0.002	2.22 (0.96–5.14)	0.062

Model 1: unadjusted; Model 2: adjusted for age, occlusion site, baseline National Institutes of Health Stroke Scale (NIHSS), hypoperfusion intensity ratio (HIR), and the time from stroke onset to revascularization (OTR).

*SHR*, stress hyperglycemia ratio; *mRS*, modified Rankin Scale; *aOR*, adjusted odds ratio; *CI*, confidence interval.

a SHR tripartite: T1 (≤0.93), T2 (0.93–1.13), T3 (≥1.13).

### Mediation and sensitivity analyses

**Figure 2:**
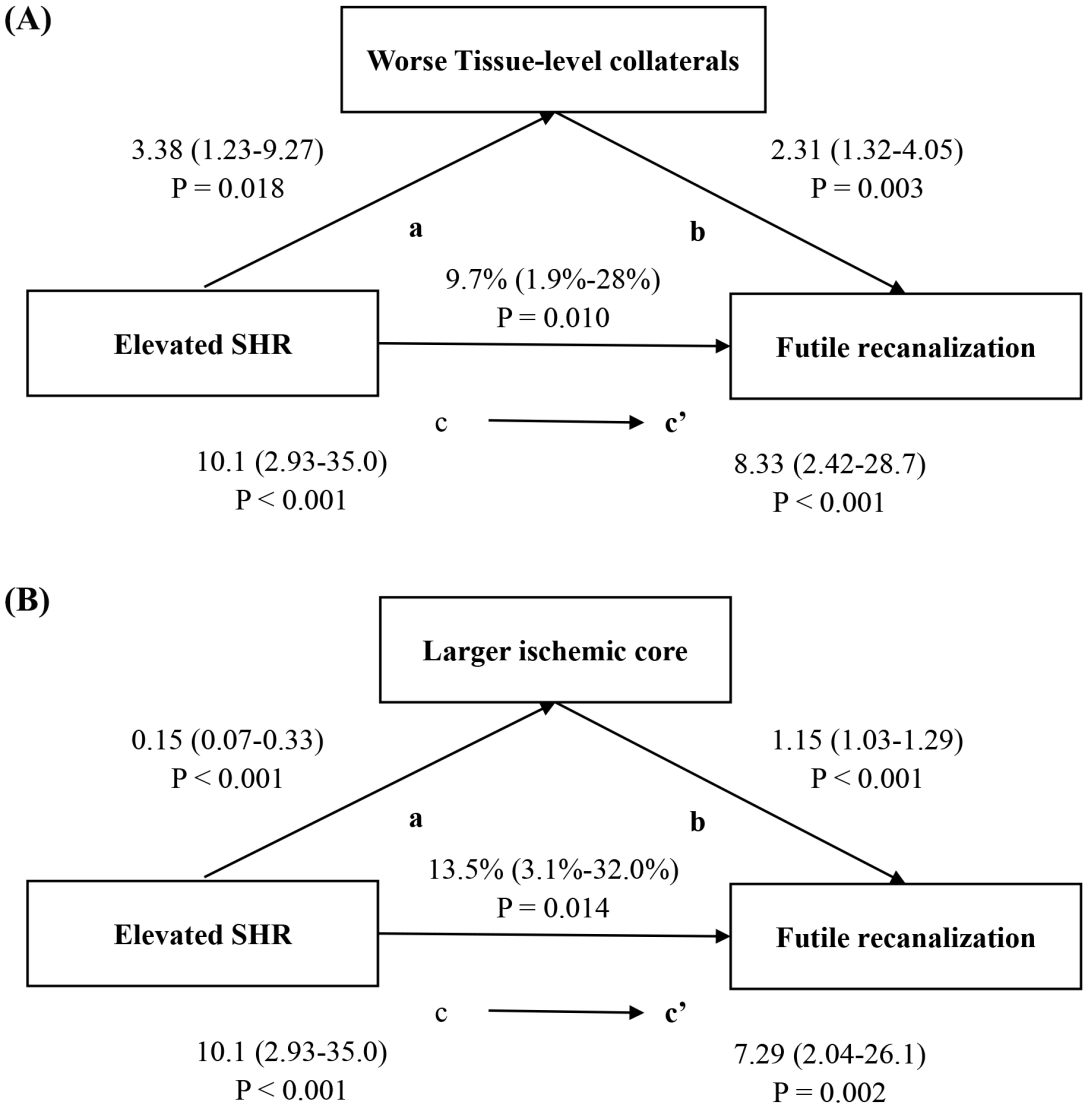
Explained proportions in the mediation analyses of the effect of the stress hyperglycemia ratio (SHR) on futile recanalization mediated by **(A)** the hypoperfusion intensity ratio (HIR) and **(B)** the baseline Alberta Stroke Program Early CT Score (ASPECTS). The odds ratios of the regression equations for each step (steps a, b, c, and c′) and the percentage of indirect effects mediated by the HIR and baseline ASPECTS are described. Binary logistic regression analysis and linear regression were adjusted for age, occlusion site, baseline National Institutes of Health Stroke Scale (NIHSS), and the time from stroke onset to revascularization (OTR). *mRS*, modified Rankin Scale.

**Figure 3:**
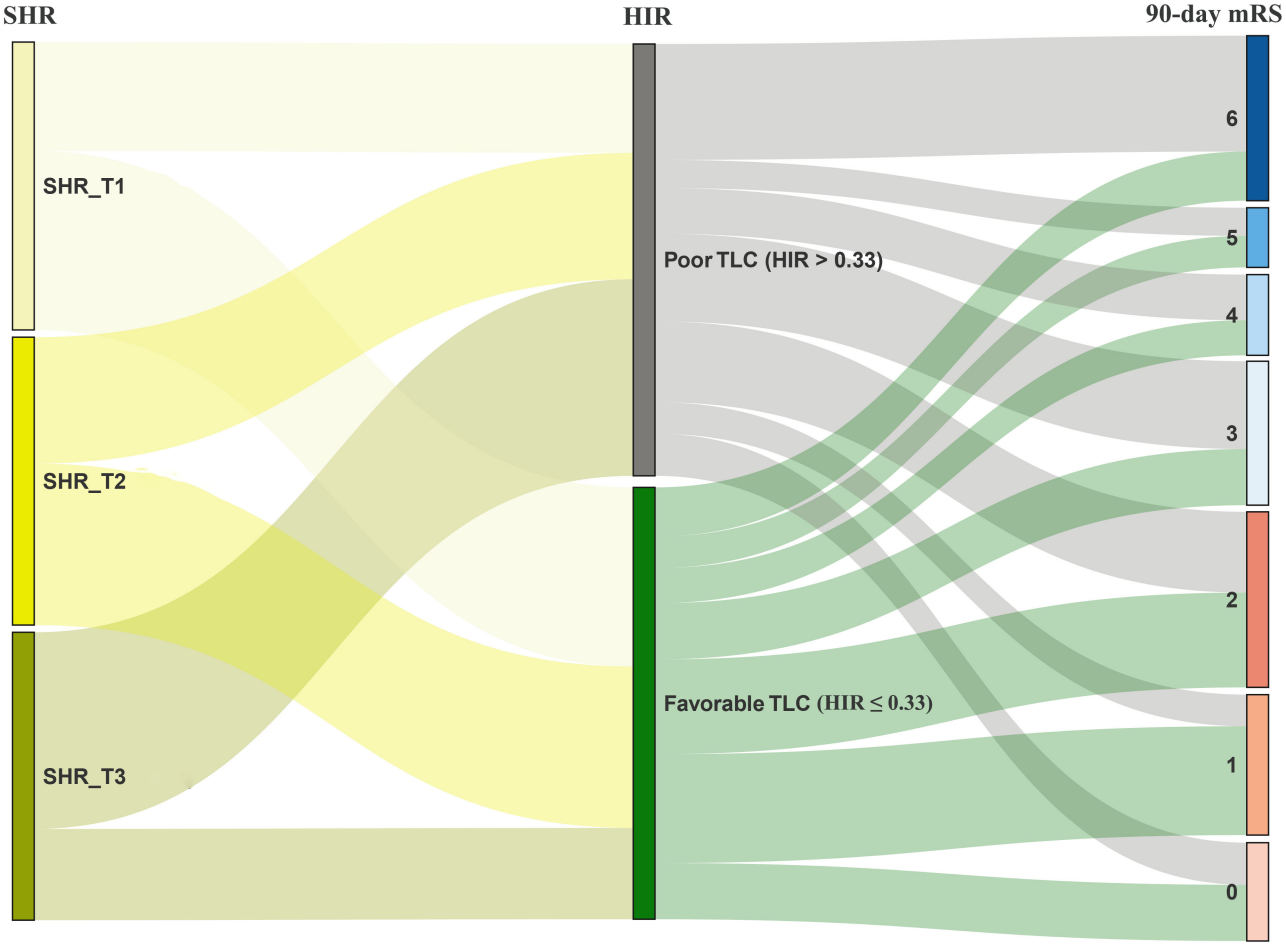
Sankey plot of the taxonomic data changes with the breadth of the tripartite stress hyperglycemia ratio (SHR) (*left side*) and hypoperfusion intensity ratio (HIR) (*middle*) levels during the 90-day modified Rankin Scale (mRS) 0–6 (*right side*). The *color* and the *width of the branches* represent the flow of specific risk factors. SHR tripartite: T1 (≤0.93), T2 (0.93–1.13), T3 (≥1.13).

We performed mediation analyses to further assess the association among SHR, the candidate mediators, and futile recanalization. In **Table 4**, the TLC (pathway a: aOR = 3.38, 95%CI = 1.23–9.27, *p* = 0.018) was worse in patients with elevated SHR and also acted as an independent predictor of futile recanalization (pathway b: aOR = 2.31, 95%CI = 1.32–4.05, *p* = 0.003). The mediation analyses showed a partial mediation effect of TLC. An elevated SHR was associated with worse TLC, accounting for 9.7% (95%CI = 1.9%–28.0%) of the harmful effect on futile recanalization (**Figure 2A**). In addition, **Table 4** shows that the baseline ASPECTS (pathway a: aOR = 0.15, 95%CI = 0.07–0.33, *p* < 0.001) was higher in patients with an elevated SHR and also acted as an independent predictor of futile recanalization (pathway b: aOR = 1.15, 95%CI = 1.03–1.29, *p* = 0.016). The mediation analyses also indicated a partial mediation effect of a larger ischemic core. An elevated SHR was associated with a larger ischemic core, accounting for 13.5% (95%CI = 3.1%–32.0%) of the harmful effect on futile recanalization (**Figure 2B**). The predominant data flow that occurred during the disease progression at the SHR and outcome levels was visualized in a Sankey plot (**Figure 3**).

**Table 4:** Regression analysis for mediation step a and step b in patients with stress hyperglycemia ratio (SHR).

Candidate mediators	Pathway a		Pathway b		Pathway c		Pathway c′	
	aOR (95%CI)^a^	*p*-value	aOR (95%CI)^a^	*p*-value	aOR (95%CI)^a^	*p*-value	aOR (95%CI)^a^	*p*-value
ASITN/SIR score	1.03 (0.47–2.27)	0.940	0.93 (0.72–1.21)	0.597	10.1 (2.93–35.0)	<0.001	10.0 (2.91–34.7)	<0.001
HIR	3.38 (1.23–9.27)	0.018	2.31 (1.32–4.05)	0.003	10.1 (2.93–35.0)	<0.001	8.33 (2.42–28.7)	<0.001
Baseline ASPECTS	0.15 (0.07–0.33)	<0.001	1.15 (1.03–1.29)	0.016	10.1 (2.93–35.0)	<0.001	7.29 (2.04–26.1)	0.002

Pathway a represents the regression coefficient of the association between the SHR index and the candidate mediators. Pathway b represents the regression coefficient of the association between the candidate mediators and futile recanalization (90-day mRS 3–6). Pathway c or c′ represents the regression coefficient of the association between SHR and futile recanalization.

*ASITN/SIR*, American Society of Interventional and Therapeutic Neuroradiology/Society of Interventional Radiology; *HIR*, hypoperfusion intensity ratio; *aOR*, adjusted odds ratio; *CI*, confidence interval.

a Adjusted for age, occlusion site, the baseline National Institutes of Health Stroke Scale (NIHSS), and the time from stroke onset to revascularization (OTR).

Furthermore, we conducted a sensitivity analysis specifically considering all candidate mediators, including the baseline ASPECTS, ASITN/SIR score, and HIR, with the association also remaining consistent between SHR and futile recanalization (**Supplementary Table S3**).

## Discussion

In this study among patients with AIS-LVO, it was found that an elevated SHR was strongly associated with a poor TLC profile and futile recanalization following EVT. This study provides important insights into impaired cerebral microvascular perfusion in patients with AIS-LVO, as we presented a mediation pathway to assess the impact of post-stroke stress hyperglycemia on the TLC and clinical outcomes.

Researchers discovered the harmful effect of hyperglycemia on the outcomes (17), but focused on post-stroke hyperglycemia management strategies and therapeutic approaches. The association between acute-phase glycemic control of insulin and clinical outcomes has also been investigated (4, 6, 7). Glycemic management strategies during the acute phase have garnered significant clinical and research attention. In recent years, studies have established consistent associations between stress hyperglycemia and clinical outcomes across acute critical conditions, including ischemic stroke and myocardial infarction (9, 18). In this study, SHR, calculated using the fasting blood glucose and HbA1c at admission, was associated with futile recanalization. The higher the SHR among patients with AIS-LVO, the worse are the functional outcomes, which is in favor with some previous studies (8, 9, 12, 19, 20). SHR is expected to replace random or fasting glucose concentration as a novel prognostic indicator and a potential therapeutic target (21).

Stress hyperglycemia refers to a transient elevation of the blood glucose levels in response to acute illness, typically returning to baseline after resolution (9). This phenomenon is prevalent among stroke patients, including those without preexisting diabetes (10). Stress hyperglycemia promotes the release of neuroinflammatory mediators, neurotoxins, and vasoconstrictive factors, which exacerbate endothelial dysfunction, impair the vascular repair mechanisms, and consequently increase the risks of futile recanalization and mortality (22–24). In addition, as a consequence of high oxidative stress, the microvasculature structures and tight junctions compromise their functionally, the infarct volume expands, and brain edema is exacerbated (25–28). While previous studies have discussed several pathophysiological mechanisms linking stress hyperglycemia to poor clinical outcomes, their focus has largely overlooked the microcirculatory and perfusion-related effects (8, 9, 12).

Our study is novel in its exploration of the association between stress hyperglycemia and cerebral perfusion and collaterals on the tissue level, further demonstrating its cause mediation for futile recanalization. Specifically, a higher SHR indicates an unfavorable TLC and a worse functional prognosis. Our clinical observations in patients with AIS-LVO suggest a link between acute glycemic stress and microcirculatory failure. The underlying mechanisms, while not directly examined here, may involve hyperglycemia-induced microvascular endothelial dysfunction. We hypothesize that the generation of toxic glucose metabolites within the endothelial cells induces cytotoxic effects, a process supported by indirect evidence from *in vitro* studies (29). Furthermore, our CTP findings in humans are corroborated by rodent experiments showing that post-stroke hyperglycemia impairs collateral perfusion recruitment and reduces salvageable tissues (30). Our results also help contextualize the negative findings from prior glucose-lowering trials, such as the INSULINFARCT trial, which found that intensive insulin therapy (targeting absolute glucose) is associated with a larger infarct growth (6). The discrepancy from our study underscores a critical distinction between absolute hyperglycemia and stress hyperglycemia. We posit that SHR outperforms absolute glucose because it more accurately reflects the severity of the acute physiological stress response by accounting for an individual’s preexisting glycemic baseline (via HbA1c) (31). Absolute glucose levels can be elevated due to chronic diabetes or acute stress, but only the latter—quantified by SHR—is likely to be tightly coupled to the catecholamine and cytokine surge that directly drives microvascular thrombo-inflammation and collateral dysfunction. Therefore, targeting the specific pathological pathway of stress-induced hyperglycemia, rather than the glucose levels per se, might be a more fruitful therapeutic strategy.

A number of limitations of this study need to be acknowledged. The retrospective design is inherently susceptible to unmeasured confounding, and despite our efforts to adjust for known prognostic variables, residual confounding may persist. Furthermore, the generalizability of our results may be primarily applicable to comprehensive stroke centers with extensive experience in EVT and with routine access to advanced perfusion imaging (CTP), which may not reflect the outcomes in all clinical settings. Secondly, the SHR index was measured only at baseline, and changes in the SHR index during the follow-up period were not assessed, leaving out the potential impact of these changes on the TLC and functional outcomes. Thirdly, the causal interpretation of our mediation analysis was limited by the observational study design. Despite adjustment for key confounders, residual confounding by unmeasured factors cannot be ruled out. The modest proportion of mediation observed suggests that other unmeasured pathways are likely involved, and the causal interpretation of these indirect effects remains limited.

## Conclusion

In patients with AIS-LVO, an elevated SHR demonstrates robust associations with impaired TLC and futile recanalization. These findings support the idea that stress hyperglycemia, reflected by fasting blood glucose and HbA1c, may be a valuable serum biomarker for the assessment of cerebral micro-perfusion in AIS-LVO.

## Data Availability

The raw data supporting the conclusions of this article will be made available by the authors, without undue reservation.
