# Supplementary material for: Impact of stress hyperglycemia mediating tissue-level collaterals on futile recanalization in large vessel occlusion patients
Source: Front Endocrinol (Lausanne). 2025 Dec 16;16:1682343. doi: 10.3389/fendo.2025.1682343 (PMC12747967; doi:10.3389/fendo.2025.1682343)
Supplement: Supplementary file 1 [file DataSheet1.docx]

**Electronic Supplementary Material**

Impact of stress hyperglycemia mediating tissue-level collaterals on futile recanalization in large vessel occlusion patients

Catalogue

[Figure S1. A flowchart of patient selection 2](#_Toc7353)

[Table S1. Baseline characteristics according to groups categorized by futile recanalization 3](#_Toc29659)

[Table S2. Multivariable logistic regression analysis for futile recanalization 5](#_Toc14587)

[Table S3. Multivariable logistic regression analysis for futile recanalization considering for candidate mediators 6](#_Toc26104)

**Figure S1.** A flowchart of patient selection

**
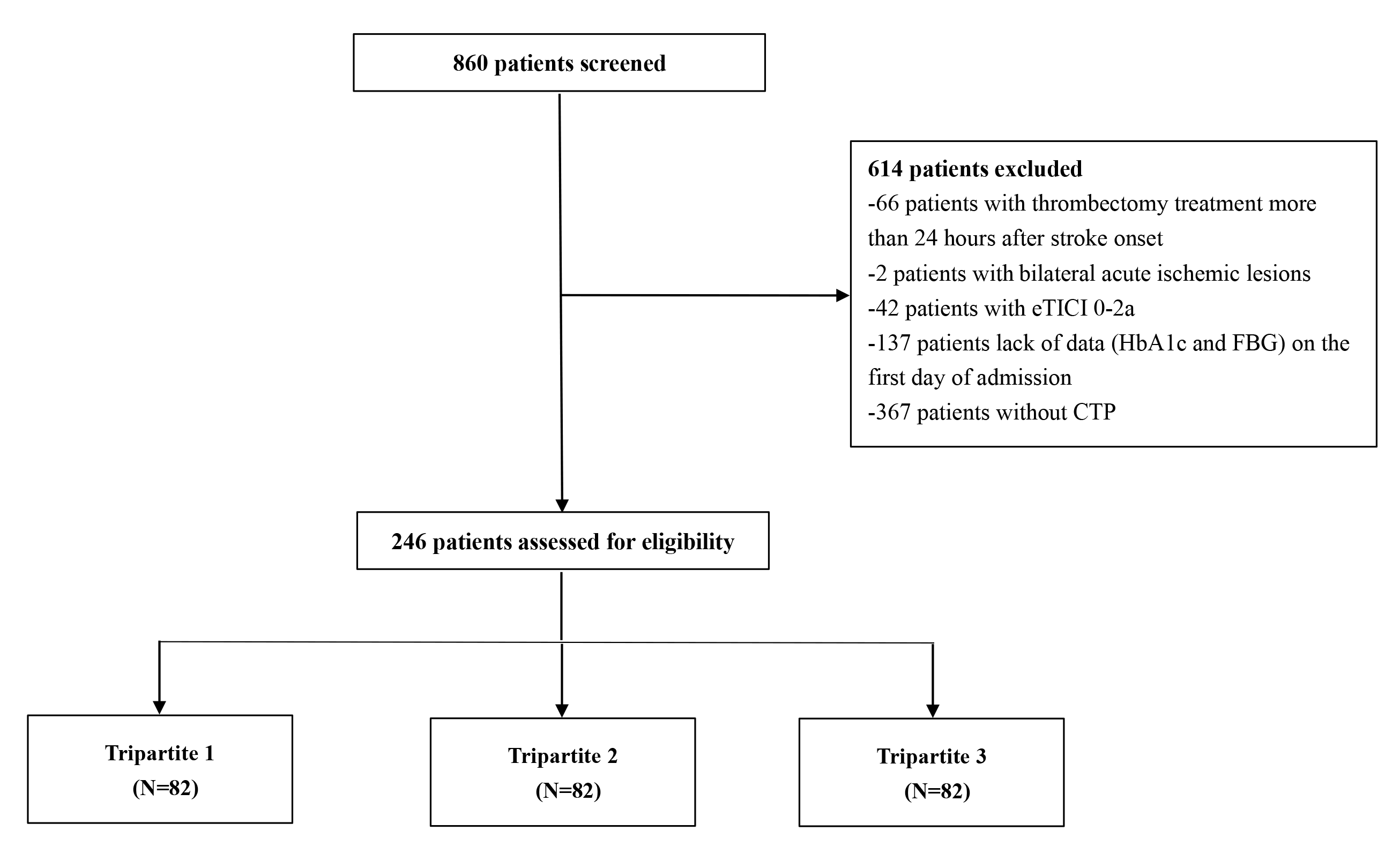
**

**Table S1.** Baseline characteristics according to groups categorized by futile recanalization (90-day mRS 3-6)

|  | **Enrolled patients** | | | **P value** |
| --- | --- | --- | --- | --- |
| **Characteristics** | **Overall**  **N=246** | **Futile recanalization**  **(90-day mRS 3-6)**  **N=128** | **Effective recanalization**  **90-day mRS 0-2**  **N=118** |  |
| Age, median (IQR) | 69 (58-76) | 72 (59-78) | 67 (57-74) | 0.006 |
| Sex/Male, n (%) | 138 (56.1) | 68 (53.1) | 70 (59.3) | 0.328 |
| Medical history, n (%) |  |  |  |  |
| Smoking | 70 (28.5) | 34 (26.6) | 36 (30.5) | 0.493 |
| Drinking | 72 (29.3) | 35 (27.3) | 37 (31.4) | 0.490 |
| Hypertension | 139 (56.5) | 74 (57.8) | 65 (55.1) | 0.666 |
| Diabetes mellitus | 42 (17.1) | 25 (19.5) | 17 (14.4) | 0.286 |
| hyperlipemia | 33 (13.4) | 14 (10.9) | 19 (16.1) | 0.235 |
| Coronary heart disease | 43 (17.5) | 24 (18.8) | 19 (16.1) | 0.585 |
| Atrial fibrillation | 96 (39.0) | 56 (43.8) | 40 (33.9) | 0.114 |
| Ischemic stroke | 32 (13.0) | 14 (10.9) | 18 (15.3) | 0.315 |
| Cerebral hemorrhage | 3 (1.2) | 2 (1.6) | 1 (0.8) | 1.000 |
| Pre-stroke mRS, median (IQR)  0  1  2 | 232 (94.3)  9 (3.7)  5 (2.0) | 121 (94.5)  5 (3.9)  2 (1.6) | 111 (94.1)  4 (3.4)  3 (2.5) | 0.915 |
| TOAST, n (%)  LAA  CE  Other | 138 (56.1)  83 (33.7)  25 (10.2) | 68 (53.1)  46 (35.9)  14 (10.9) | 70 (59.3)  37 (31.4)  11 (9.3) | 0.619 |
| Occlusion site, n (%)  ICA  MCA-M1  MCA-M2 | 79 (32.1)  126 (51.2)  41 (16.7) | 45 (35.2)  63 (49.2)  20 (15.6) | 34 (28.8)  63 (53.4)  21 (17.8) | 0.562 |
| Baseline NIHSS score, median (IQR) | 13 (10-18) | 15 (11-19) | 11 (8-16) | <0.001 |
| Baseline ASPECTS score, median (IQR) | 7 (5-8) | 6 (4-8) | 8 (6-9) | <0.001 |
| ASITN/SIR score, median (IQR) | 1 (1-2) | 1 (1-2) | 2 (1-2) | 0.232 |
| HIR, median (IQR), min | 0.33 (0.12-0.48) | 0.38 (0.22-0.55) | 0.26 (0.05-0.40) | <0.001 |
| OTP, median (IQR), min | 380 (246-581) | 394 (261-584) | 345 (234-584) | 0.408 |
| OTR, median (IQR), min | 497 (338-723) | 514 (368-722) | 475 (315-773) | 0.295 |
| Intravenous_thrombolysis, median (IQR) | 110 (44.7) | 58 (45.3) | 52 (44.1) | 0.844 |
| Glucose (mmol/L), median (IQR) | 7.1 (6.0-8.6) | 7.7 (6.6-9.6) | 6.3 (5.7-7.5) | <0.001 |
| HbA1C (%), median (IQR) | 5.8 (5.5-6.3) | 5.9 (5.5-6.4) | 5.7 (5.5-6.2) | 0.209 |
| SHR, median (IQR) | 1.03 (0.89-1.19) | 1.11 (0.94-1.29) | 0.97 (0.85-1.24) | <0.001 |

^a^ SHR tripartite: T1 (≤ 0.93), T2 (0.93-1.13), T3 (≥ 1.13)

Abbreviation: SHR, stress hyperglycemia ratio; IQR, interquartile range; TOAST, the Trial of ORG 10172 in Acute Stroke Treatment; LAA, large-artery atherosclerosis; CE, cardiogenic embolism; ICA, internal carotid artery; MCA, middle cerebral artery; NIHSS, National Institutes of Health Stroke Scale; ASITN/SIR the American Society of Interventional and Therapeutic Neuroradiology/Society of Interventional Radiology; HIR, hypoperfusion intensity ratioa; ASPECTS, Alberta Stroke Program Early CT Score; OTP, the time from stroke onset to groin puncture; OTR, the time from stroke onset to revascularization; mRS, modified Rankin Scale

**Table S2.** Multivariable logistic regression analysis for futile recanalization (90-day mRS 3-6)

| **Variables** | **Multivariable analysis** | | **Multivariable analysis** | | |
| --- | --- | --- | --- | --- | --- |
|  | **aOR (95% CI)** | **P value** | **aOR (95% CI)** | | **P value** |
| Age | 1.02 (1.00-1.05) | 0.078 | 1.02 (1.00-1.05) | 0.055 | |
| Baseline NIHSS | 1.04 (0.99-1.10) | 0.096 | 1.05 (1.00-1.11) | 0.067 | |
| Occlusion site  ICA  MCA-M1  MCA-M2 | Reference  0.85 (0.45-1.62)  0.70 (0.30-1.63) | 0.627  0.409 | Reference  0.89 (0.47-1.68)  0.67 (0.29-1.58) | 0.712  0.363 | |
| OTR | 1.00 (1.00-1.00) | 0.199 | 1.00 (1.00-1.00) | 0.725 | |
| HIR | 9.22 (2.08-40.79) | 0.003 | 8.49 (1.91-37.76) | 0.005 | |
| SHR | 7.82 (2.26-27.10) | 0.001 | —— | —— | |
| SHR tripartite^a^  T1  T2  T3 | —— | —— | Reference  1.84 (0.94-3.62)  3.56 (1.73-7.30) | 0.076  <0.001 | |

^a^ SHR tripartite: T1 (≤ 0.93), T2 (0.93-1.13), T3 (≥ 1.13)

Abbreviation: SHR, stress hyperglycemia ratio; aOR, adjusted odds ratio; CI, confidence interval; LAA, large-artery atherosclerosis; CE, cardiogenic embolism; ICA, internal carotid artery; MCA, middle cerebral artery; NIHSS, National Institutes of Health Stroke Scale; HIR, hypoperfusion intensity ratioa; OTR, the time from stroke onset to revascularization

**Table S3.** Multivariable logistic regression analysis for futile recanalization (90-day mRS 3-6) considering for candidate mediators

| **Variables** | **Multivariable analysis** | | **Multivariable analysis** | |
| --- | --- | --- | --- | --- |
|  | **aOR (95% CI)** | **P value** | **aOR (95% CI)** | **P value** |
| Age | 1.03 (1.01-1.05) | 0.043 | 1.03 (1.01-1.06) | 0.029 |
| Baseline NIHSS | 1.04 (0.98-1.09) | 0.176 | 1.04 (0.99-1.10) | 0.138 |
| Baseline ASPECTS | 0.86 (0.76-0.98) | 0.024 | 0.86 (0.76-0.97) | 0.016 |
| Occlusion site  ICA  MCA-M1  MCA-M2 | Reference  0.86 (0.44-1.69)  0.77 (0.32-1.86) | 0.658  0.559 | Reference  0.88 (0.44-1.73)  0.74 (0.31-1.81) | 0.703  0.512 |
| ASITN/SIR score | 0.98 (0.74-1.29) | 0.876 | 0.97 (0.73-1.29) | 0.849 |
| OTR | 1.00 (1.00-1.00) | 0.199 | 1.00 (1.00-1.00) | 0.147 |
| HIR | 6.35 (1.78-22.62) | 0.019 | 5.64 (1.19-26.68) | 0.029 |
| SHR | 6.35 (1.78-22.62) | 0.004 | —— | —— |
| SHR tripartite^a^  T1  T2  T3 | —— | —— | Reference  1.81 (0.92-6.54)  3.15 (1.52-6.54) | 0.088  0.002 |

^a^ SHR tripartite: T1 (≤ 0.93), T2 (0.93-1.13), T3 (≥ 1.13)

Abbreviation: SHR, stress hyperglycemia ratio; aOR, adjusted odds ratio; CI, confidence interval; LAA, large-artery atherosclerosis; CE, cardiogenic embolism; ICA, internal carotid artery; MCA, middle cerebral artery; NIHSS, National Institutes of Health Stroke Scale; ASITN/SIR the American Society of Interventional and Therapeutic Neuroradiology/Society of Interventional Radiology; HIR, hypoperfusion intensity ratioa; ASPECTS, Alberta Stroke Program Early CT Score; OTR, the time from stroke onset to revascularization
